# Supplementary material for: Curcumin Suppresses TGF-β1-Induced Myofibroblast Differentiation and Attenuates Angiogenic Activity of Orbital Fibroblasts
Source: Int J Mol Sci. 2021 Jun 25;22(13):6829. doi: 10.3390/ijms22136829 (PMC8268269; doi:10.3390/ijms22136829)
Supplement: Supplementary file 1 [file ijms-22-06829-s001.zip › Supplementary Figures Rev2 0615.pdf]

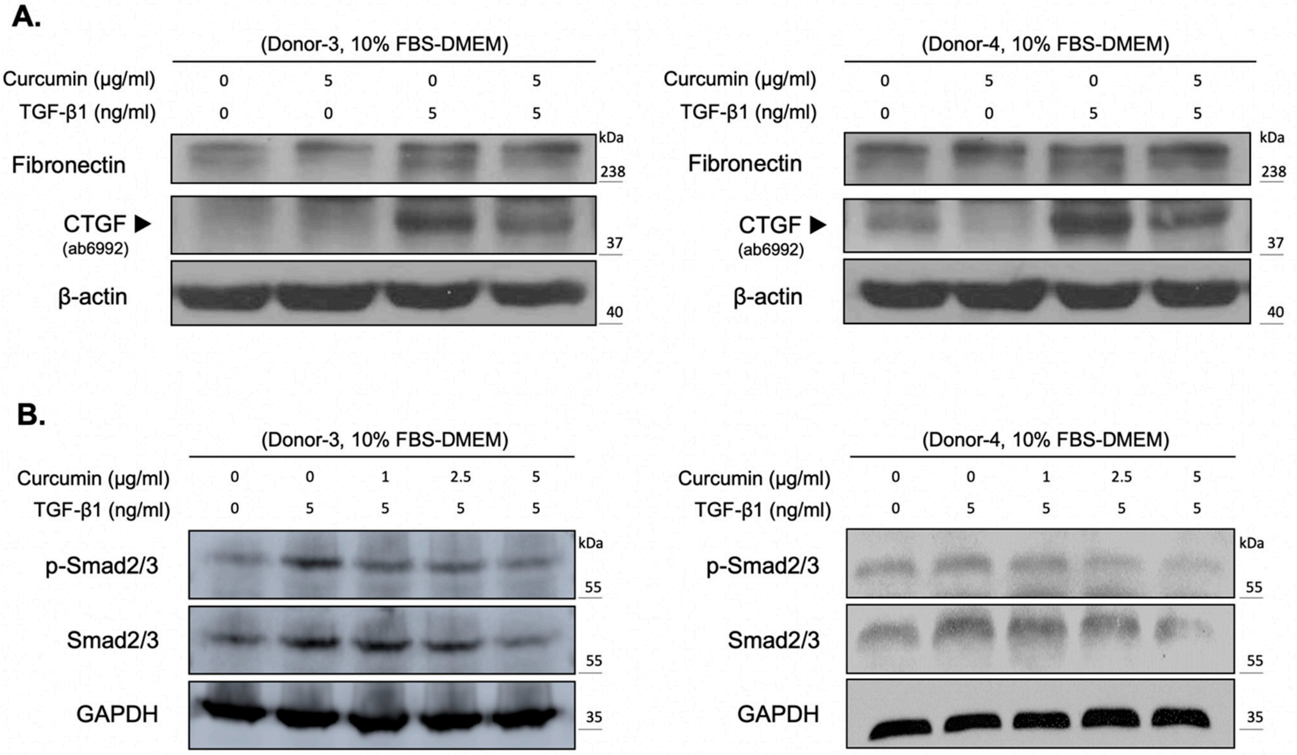

Figure S1. Curcumin suppresses the TGF-β1-activated myofibroblast differentiation and TGF-β1 signaling in GO orbital fibroblasts (Donor 3 and Donor 4). (**A**). Western blots showed the expression levels of CTGF and Fibronectin at indicated culture conditions of the orbital fibroblasts from donor 3 and donor 4. Cells were seeded in 10% FBS-containing medium overnight and treated with curcumin for 1 hour, followed by the addition of TGF-β1 for another 24 hours. (**B**). Western blots showed the expression of phosphorylated Smad 2/3 (p-Smad 2/3) and total Smad proteins (Smad 2/3) at indicated conditions of the orbital fibroblasts from donor 3 and donor 4. Cells were seeded in 10% FBS-containing medium overnight and pretreated with the indicated dose of curcumin for 60 minutes, followed by the addition of TGF-β1 for another 60 minutes.

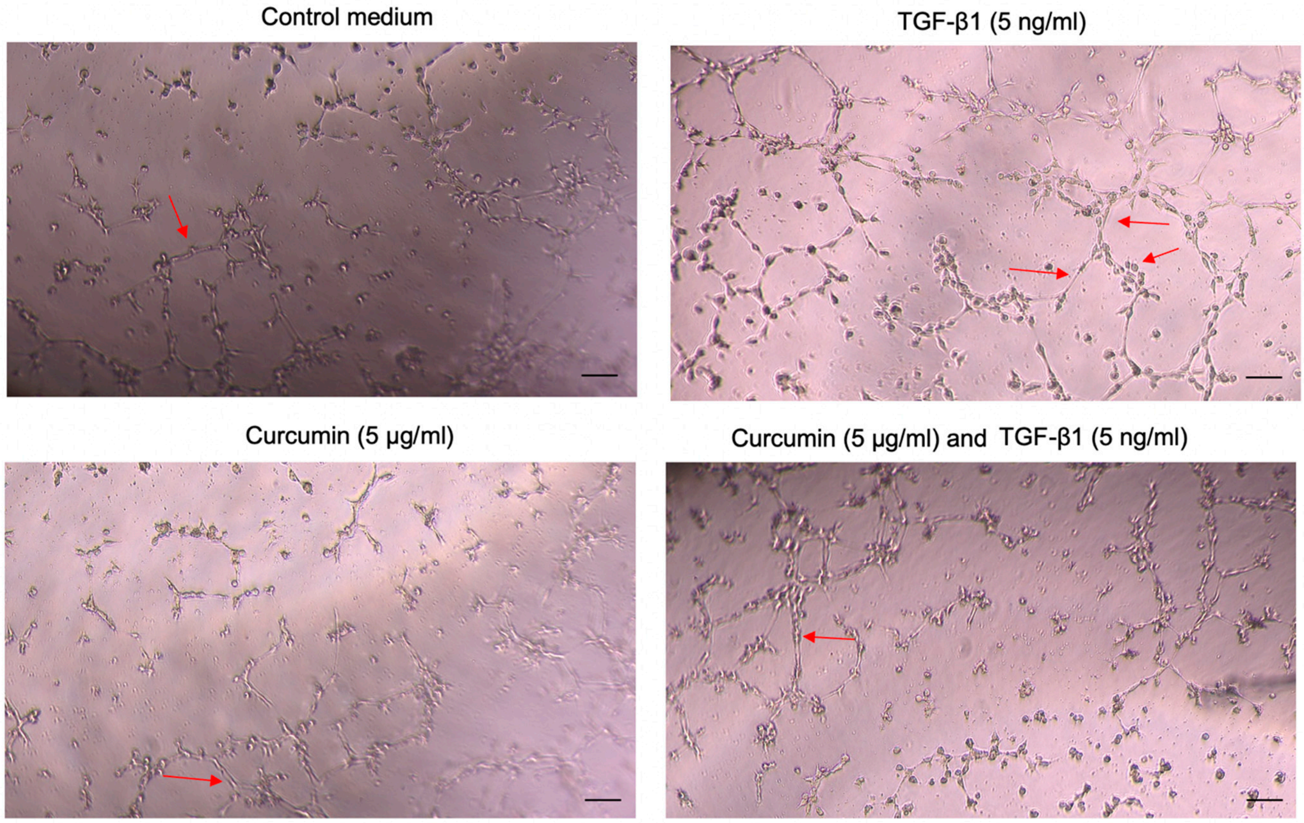

Figure S2. Curcumin decreased the TGF-β1-induced tube-forming capacity of EA. hy926 endothelial cells. Representative images showed that the tubes formed on the migratory of EA. hy926 endothelial cells were treated with an indicated conditions from Graves' orbital fibroblasts (donor 2). Scale bar is 100μm.

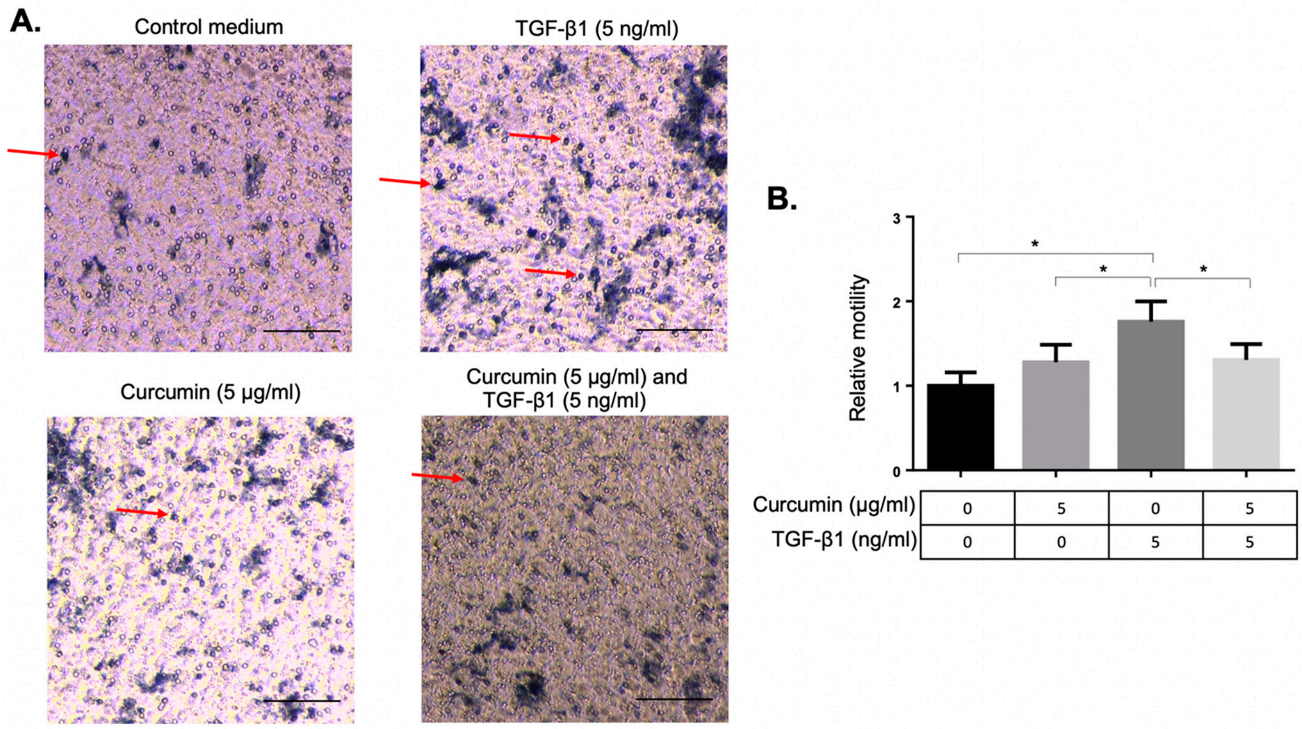

Figure S3. Curcumin attenuates the TGF-β1-induced transwell migratory ability of EA. hy926 endothelial cells. (**A**). Representative images showed the migrated EA. hy926 endothelial cells treated with indicated conditions from Graves' orbital fibroblasts (donor 2). Scale bar is 100μm. (**B**). The relative transwell migratory motility of EA. hy926 endothelial cells cultured at indicated conditioned medium from orbital fibroblasts. (10x field) Data represents as mean ± SD (n>3). *, P<0.05.

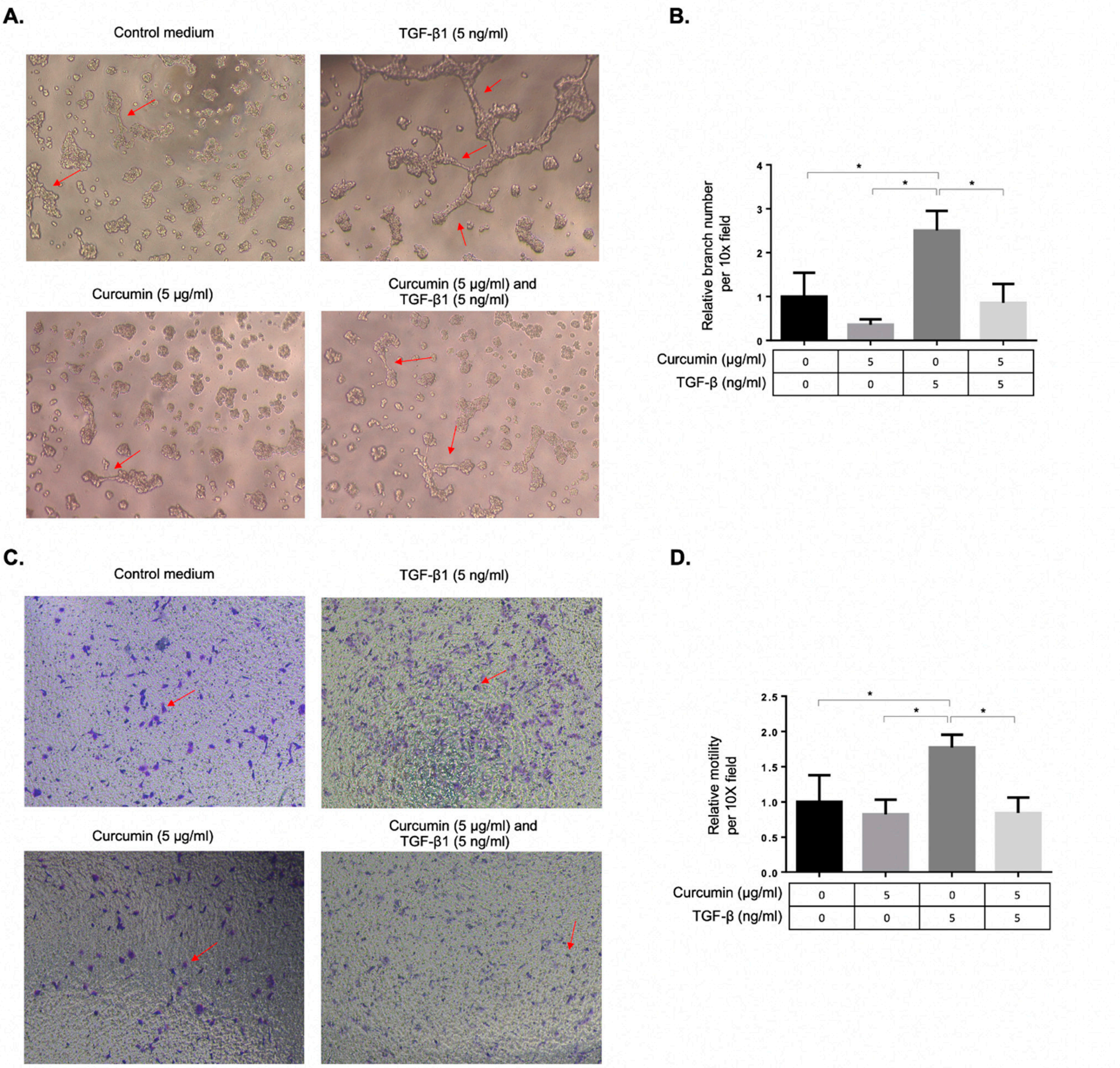

Figure S4. Curcumin attenuates the TGF-β1-induced tube-forming capacity and transwell migratory ability of HMEC-1 endothelial cells. (**A**). Representative images showed that the tubes formed on the migratory of HMEC-1 endothelial cells were treated with an indicated conditions from Graves' orbital fibroblasts (donor 2). 4x field (**B**). The relative tube-branching numbers of HMEC-1 endothelial cells cultured at indicated conditioned medium from orbital fibroblasts. (10x field) Data are expressed as mean ± SD (n=3). *, P<0.05. (**C**). Representative images showed the migrated HMEC-1 endothelial cells treated with indicated conditions from Graves' orbital fibroblasts (donor 2). 4x field. (**D**). The relative transwell migratory motility of HMEC-1 endothelial cells cultured at indicated conditioned medium from orbital fibroblasts. (10x field) Data represents as mean ± SD (n=6). *, P<0.05.
