# Supplementary material for: Curcumin Suppresses TGF-β1-Induced Myofibroblast Differentiation and Attenuates Angiogenic Activity of Orbital Fibroblasts
Source: Int J Mol Sci. 2021 Jun 25;22(13):6829. doi: 10.3390/ijms22136829 (PMC8268269; doi:10.3390/ijms22136829)
Supplement: Supplementary file 1 [file ijms-22-06829-s001.zip › Supplementary S5 uncropped blots.pdf]

Fig 1C. Exp 1

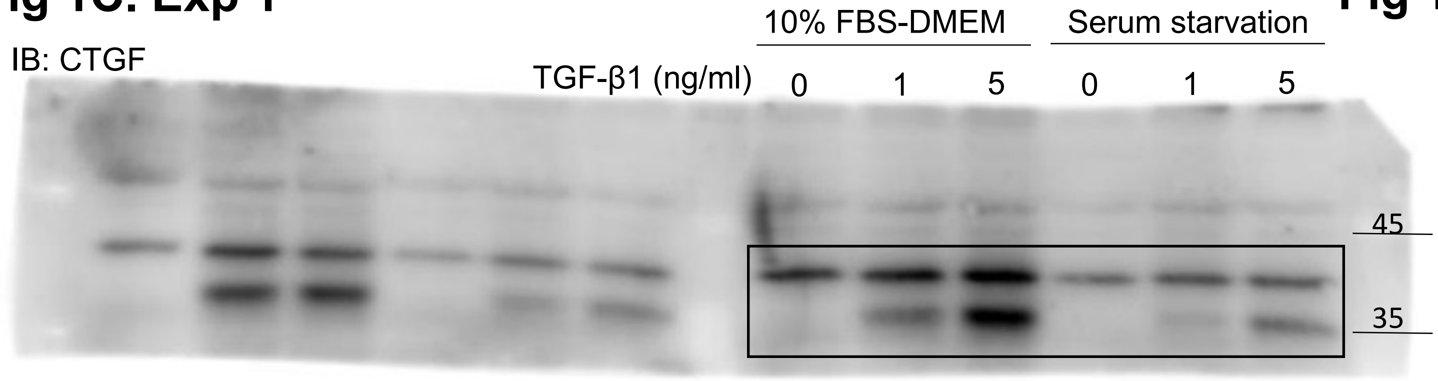

Fig 1D. Exp1

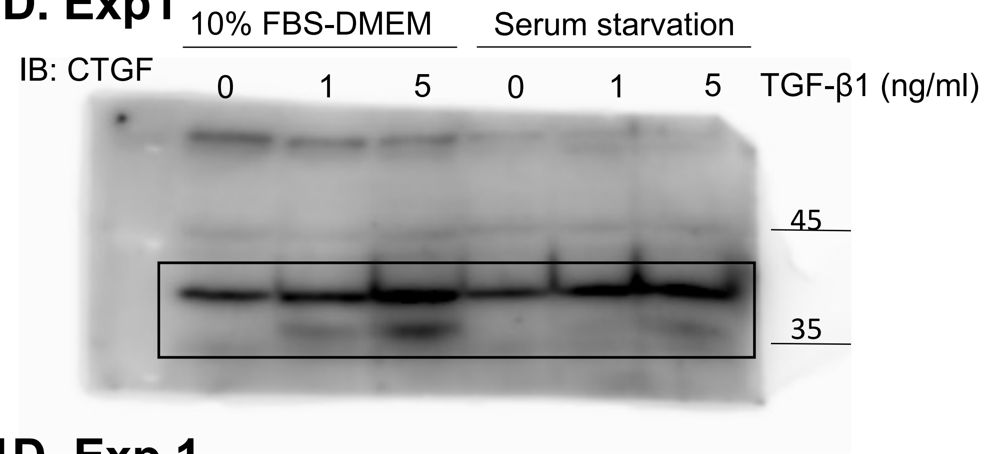

Fig 1C. Exp 1

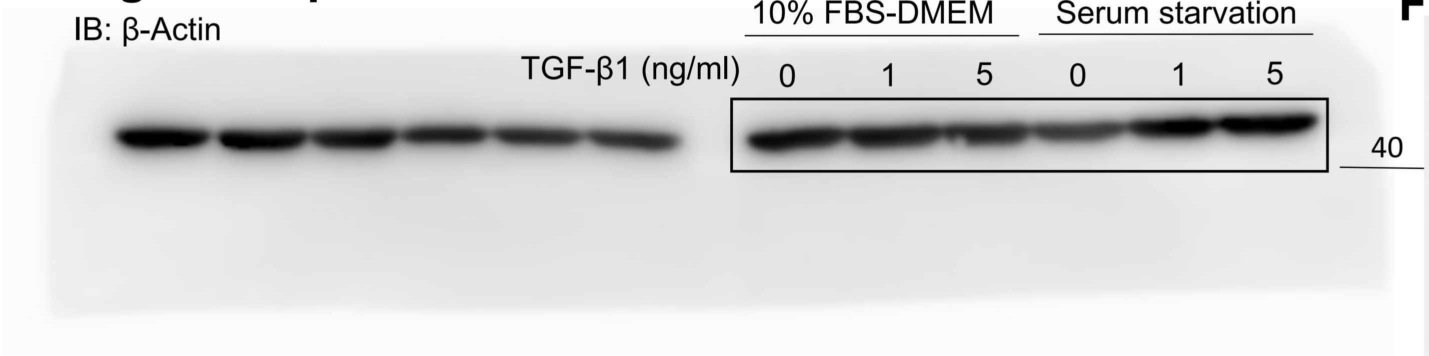

Fig 1D. Exp 1

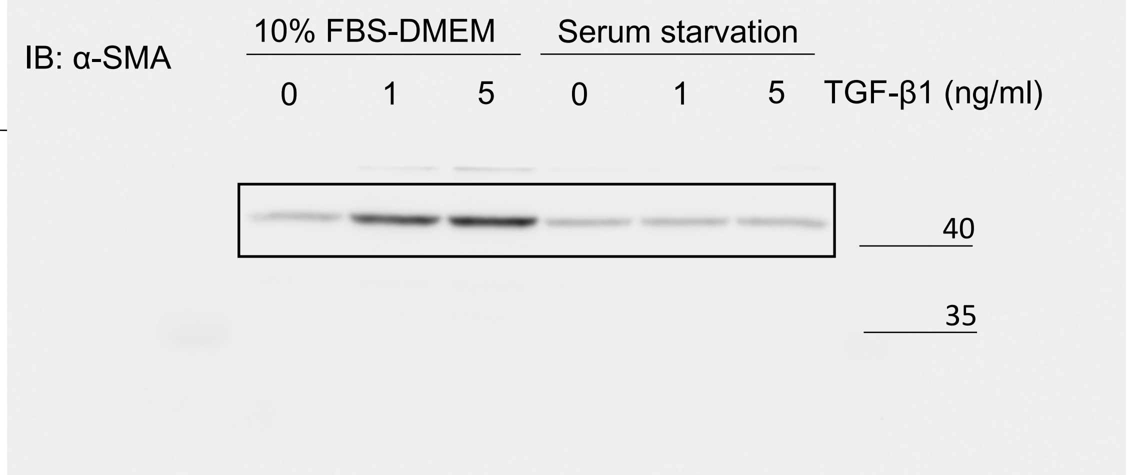

Fig 1C. Exp 1

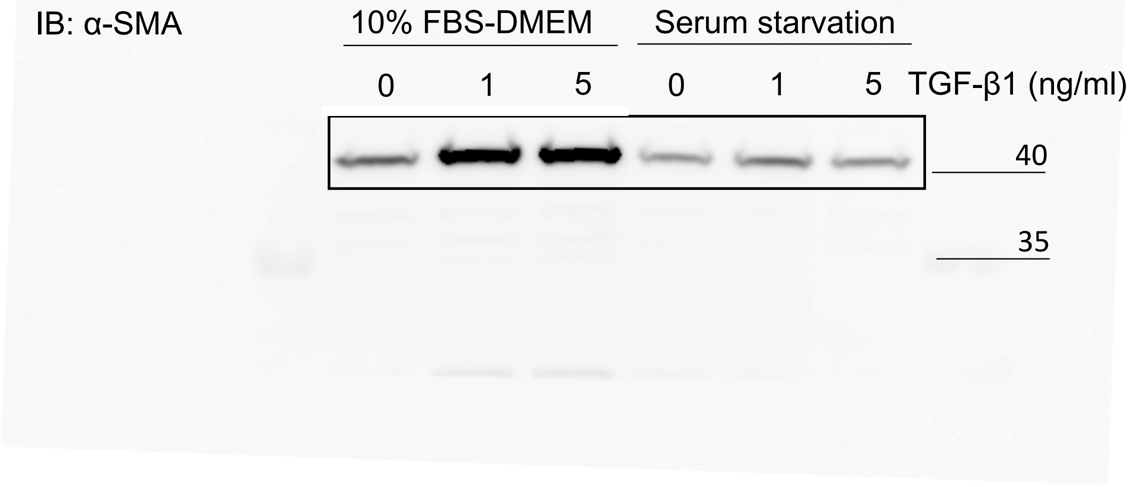

Fig 1D. Exp 1

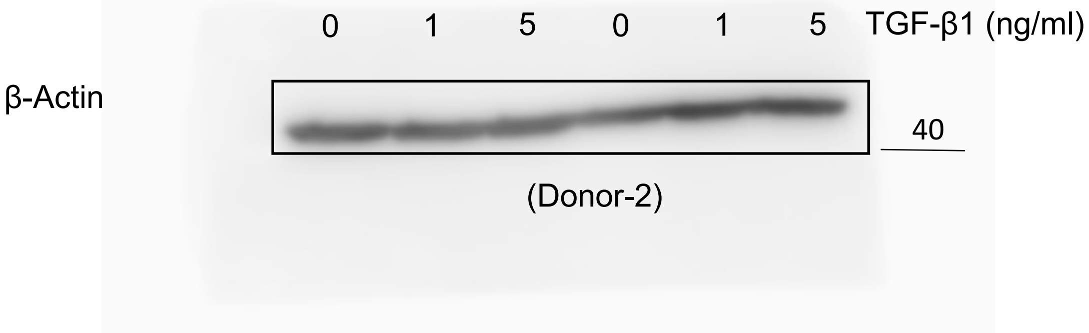

Fig 1C. Exp 2

IB: CTGF

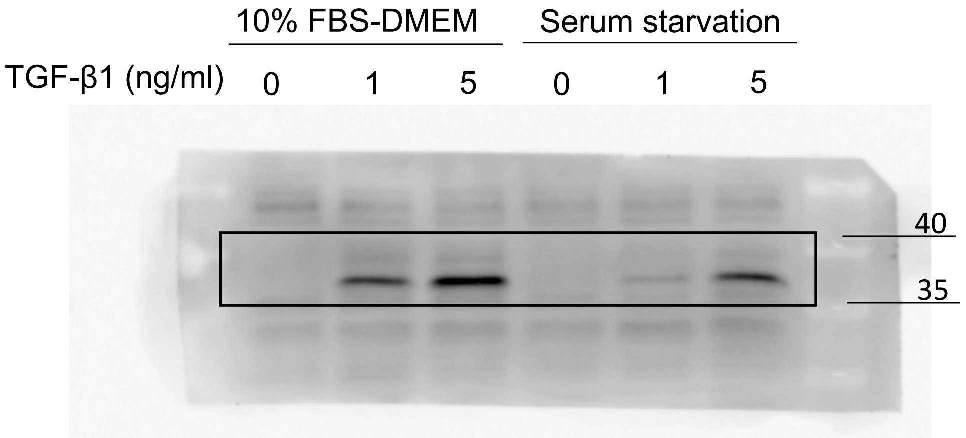

Fig 1D. Exp 2

IB: CTGF

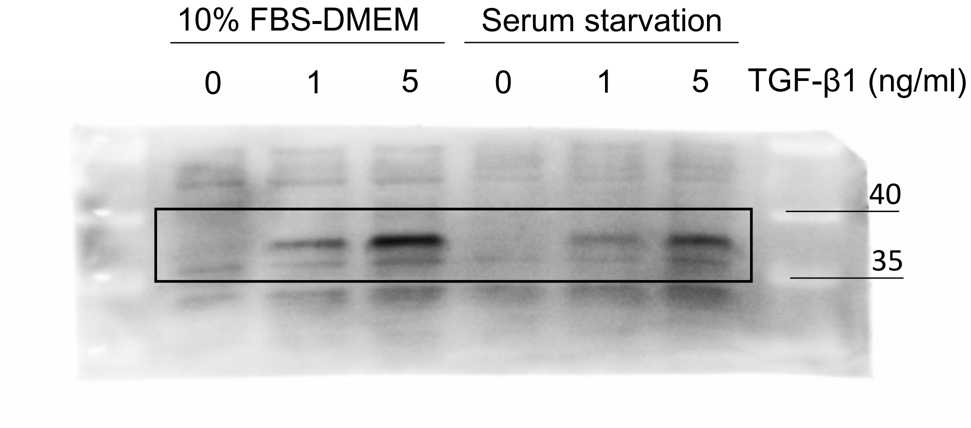

Fig 1C. Exp 2

IB: β-Actin

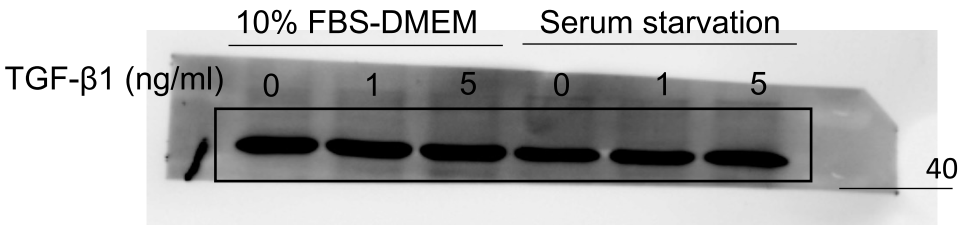

(Donor-1)

Fig 1D. Exp 2

IB: β-Actin

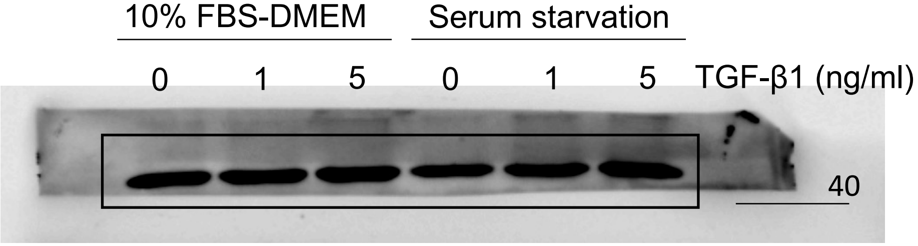

(Donor-2)

Fig 2B. Exp 1

IB: CTGF

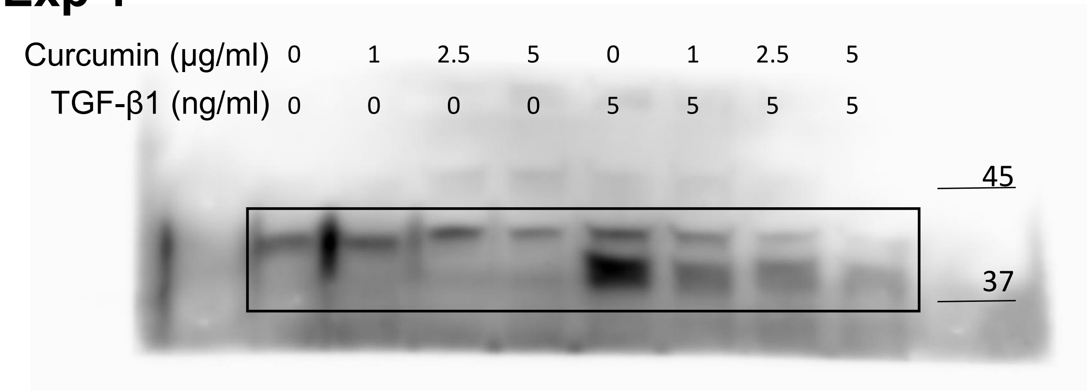

Fig 2B. Exp 1

IB: α-SMA

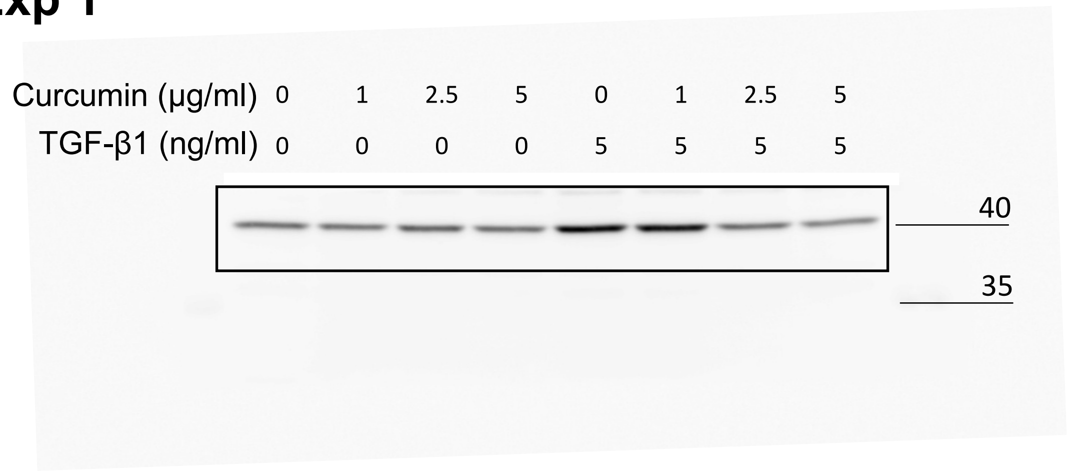

Fig 2B. Exp 1

IB: β-Actin

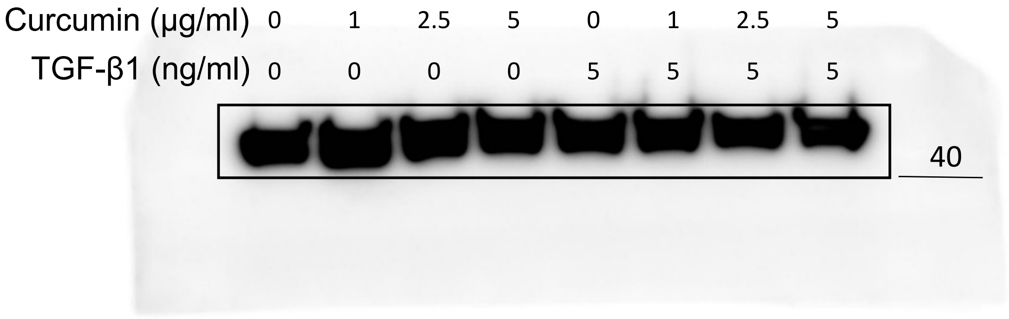

Fig 2C.

IB: CTGF

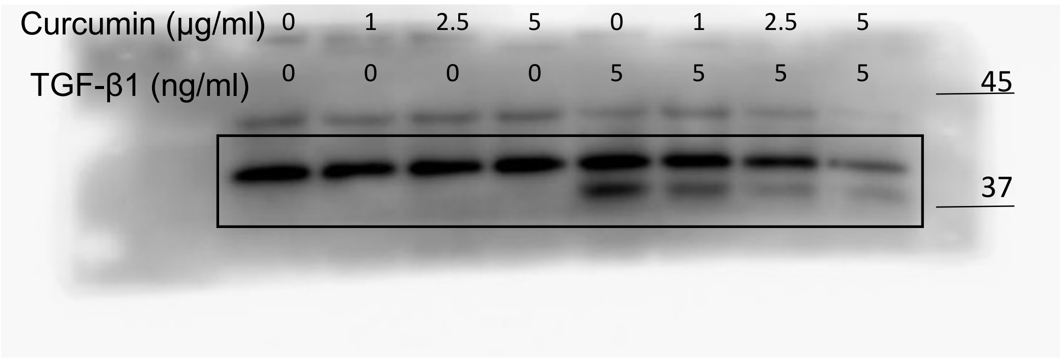

Fig 2C.

IB: α-SMA

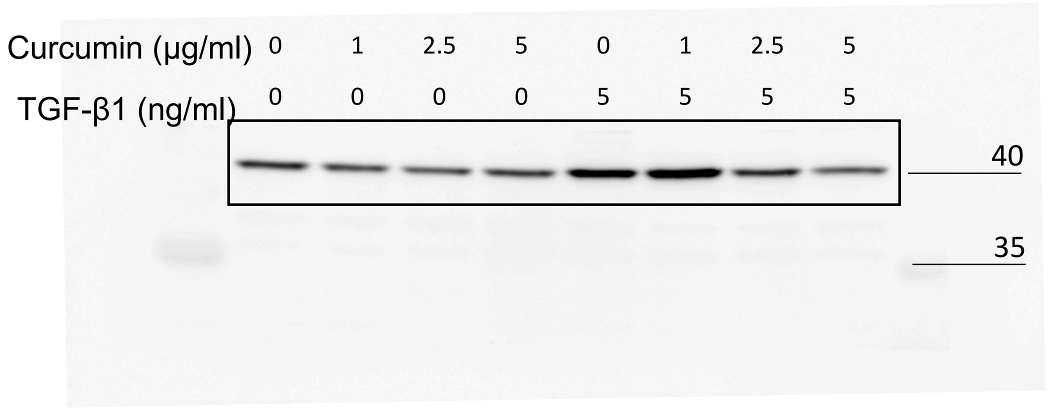

Fig 2C.

IB: β-Actin

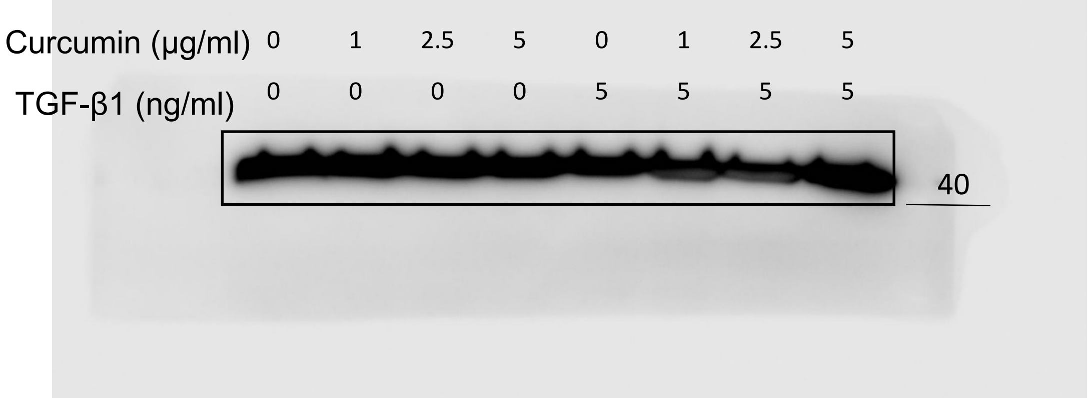

Fig 2B. Exp 2

IB: CTGF

Curcumin (μg/ml)	0	1	2.5	5	0	1	2.5	5
TGF-β1 (ng/ml)	0	0	0	0	5	5	5	5

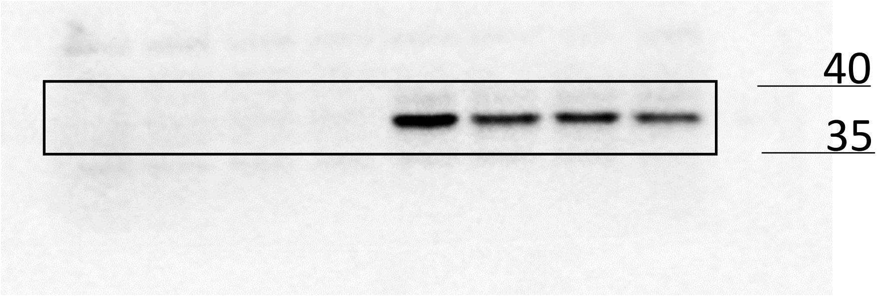

Fig 2B. Exp 2

IB: β-Actin

Curcumin (μg/ml)	0	1	2.5	5	0	1	2.5	5
TGF-β1 (ng/ml)	0	0	0	0	5	5	5	5

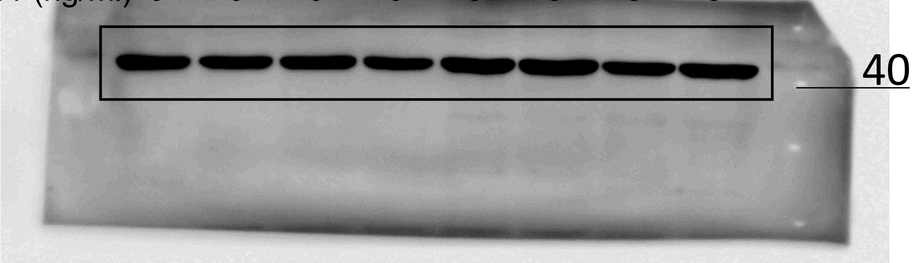

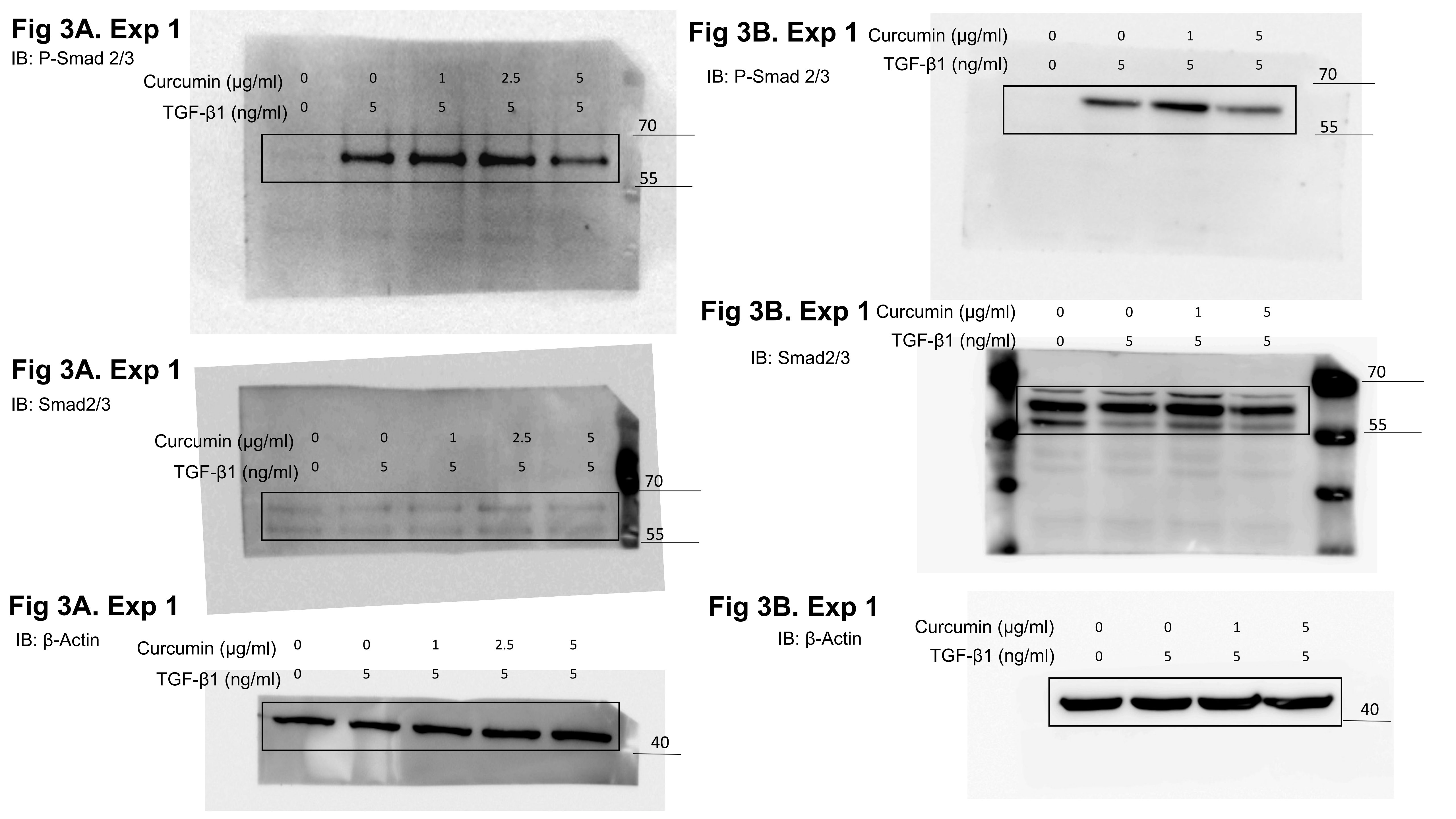

Fig 3A. Exp 1
IB: P-Smad 2/3
Curcumin (µg/ml) 0 0 1 2.5 5
TGF-β1 (ng/ml) 0 5 5 5 5
70
55
Fig 3A. Exp 1
IB: Smad2/3
Curcumin (µg/ml) 0 0 1 2.5 5
TGF-β1 (ng/ml) 0 5 5 5 5
70
55
Fig 3A. Exp 1
IB: β-Actin
Curcumin (µg/ml) 0 0 1 2.5 5
TGF-β1 (ng/ml) 0 5 5 5 5
40
Fig 3B. Exp 1
IB: P-Smad 2/3
Curcumin (µg/ml) 0 0 1 5
TGF-β1 (ng/ml) 0 5 5 5
70
55
Fig 3B. Exp 1
IB: Smad2/3
Curcumin (µg/ml) 0 0 1 5
TGF-β1 (ng/ml) 0 5 5 5
70
55
Fig 3B. Exp 1
IB: β-Actin
Curcumin (µg/ml) 0 0 1 5
TGF-β1 (ng/ml) 0 5 5 5
40

Fig 3A. Exp 2

IB: P-Smad 2/3

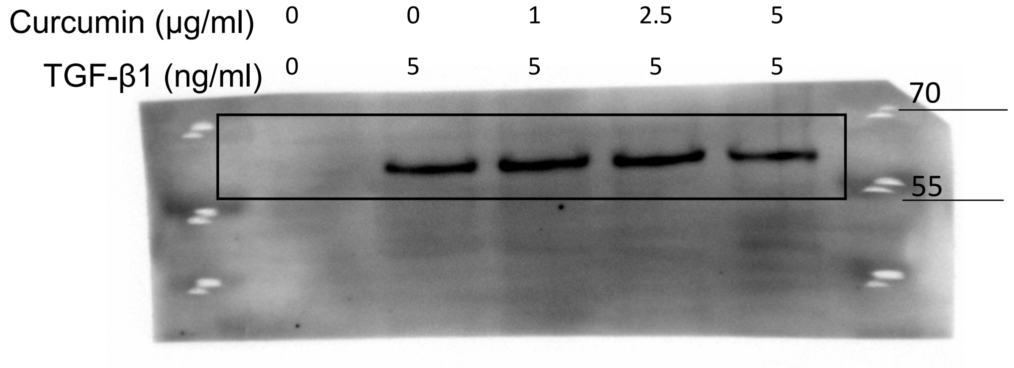

Fig 3A. Exp 2

IB: Smad2/3

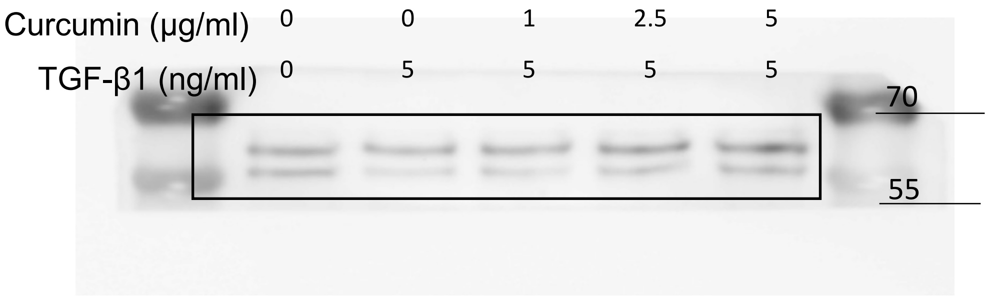

Fig 3A. Exp 2

IB: β-Actin

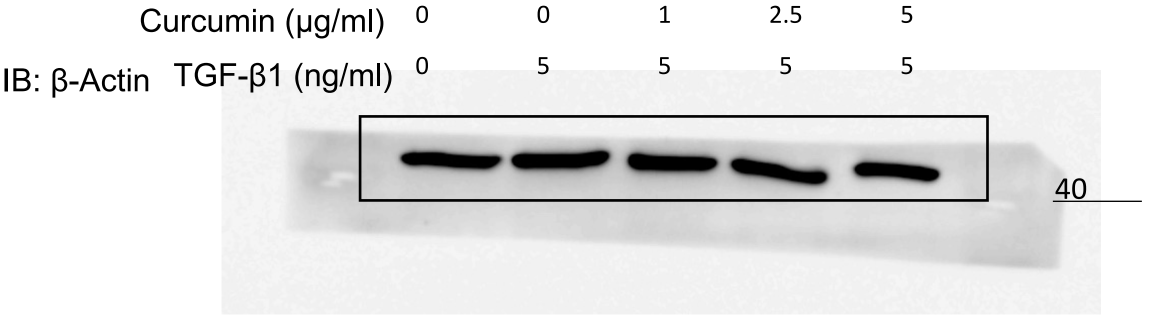

Fig 3B. Exp 2

IB: P-Smad 2/3

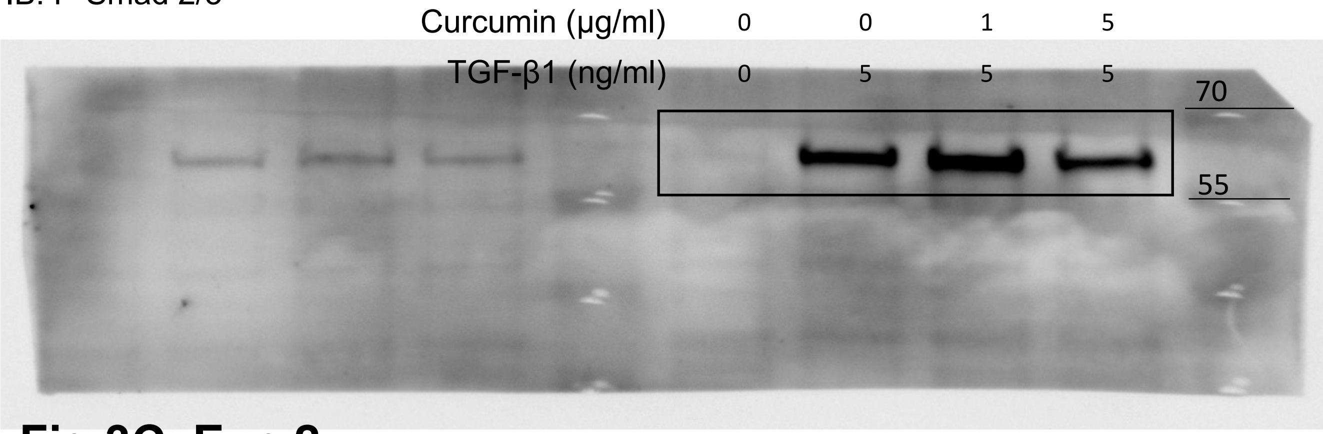

Fig 3C. Exp 2

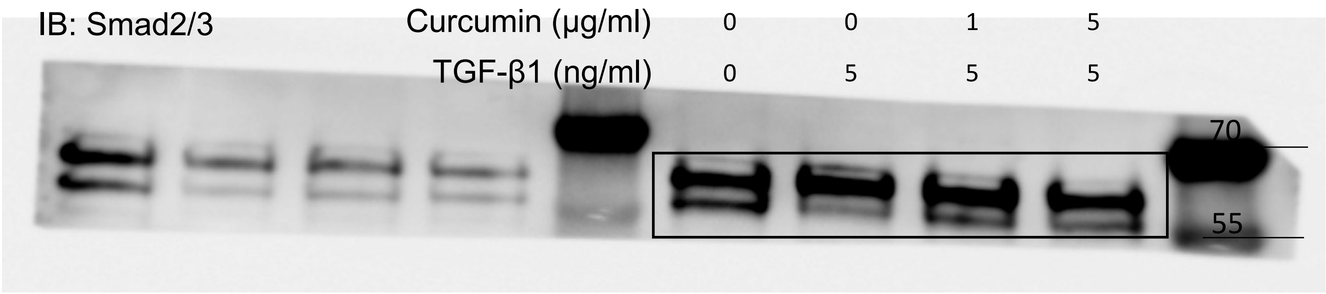

Fig 3C. Exp 2

IB: β-Actin

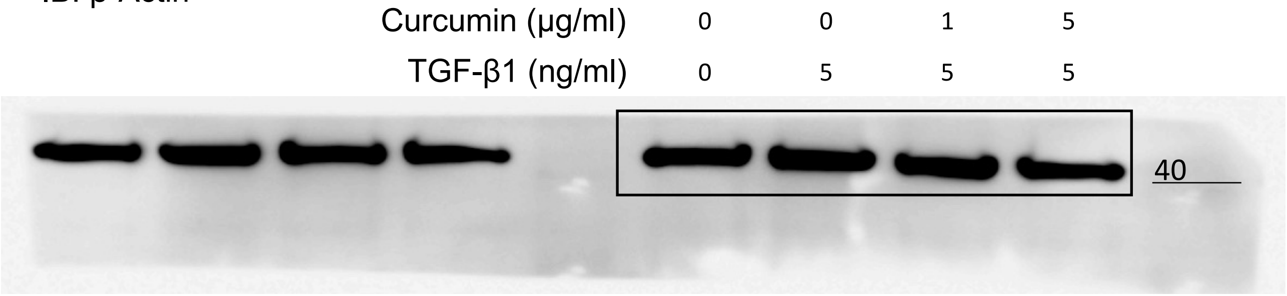

Fig S1A.

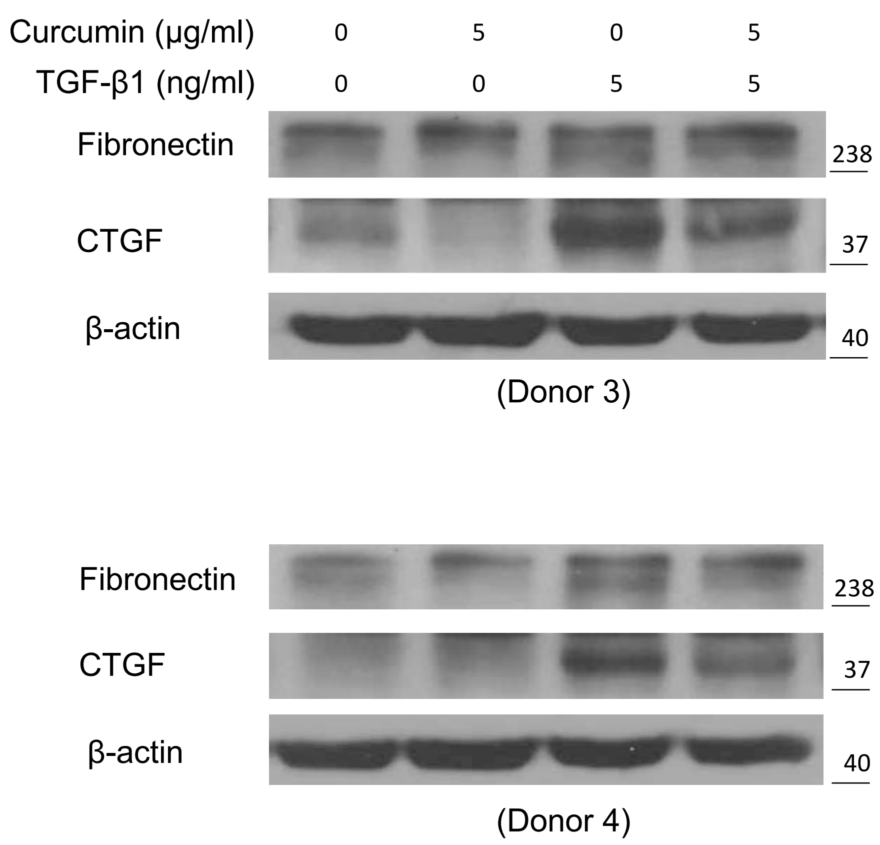

Fig S1B.

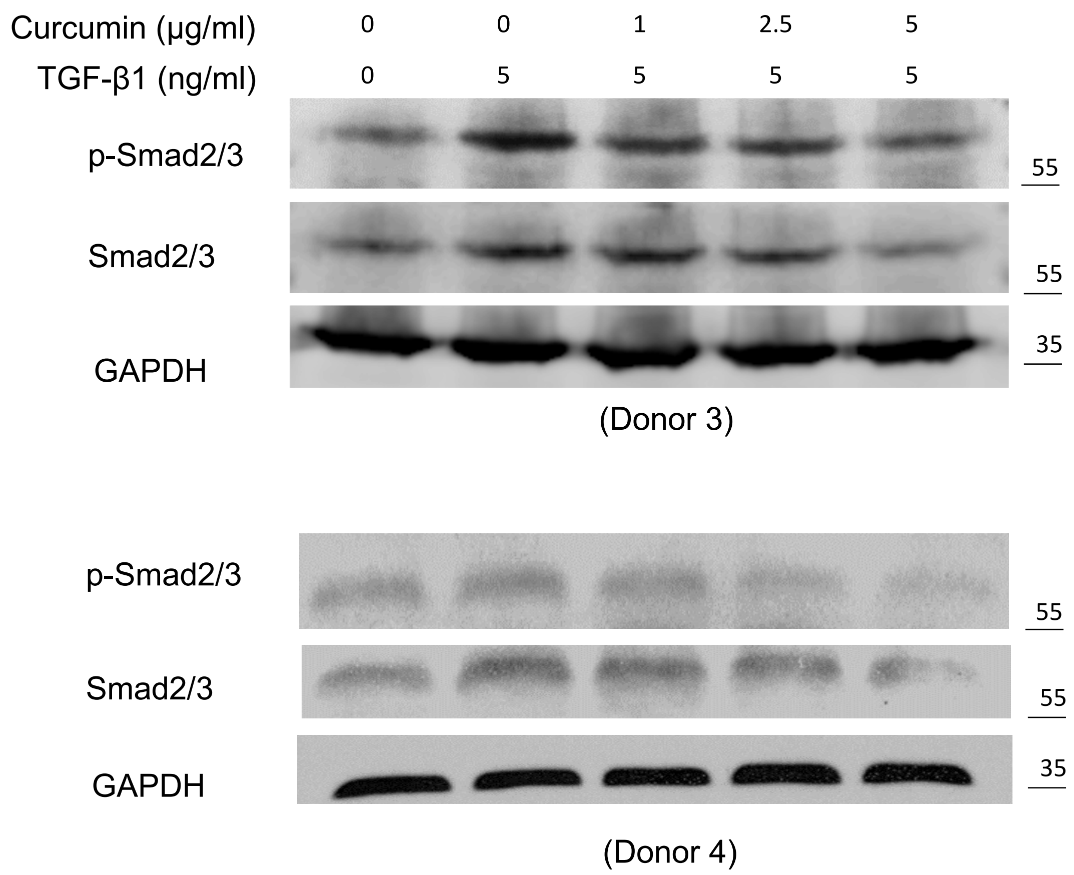

Fig S2.

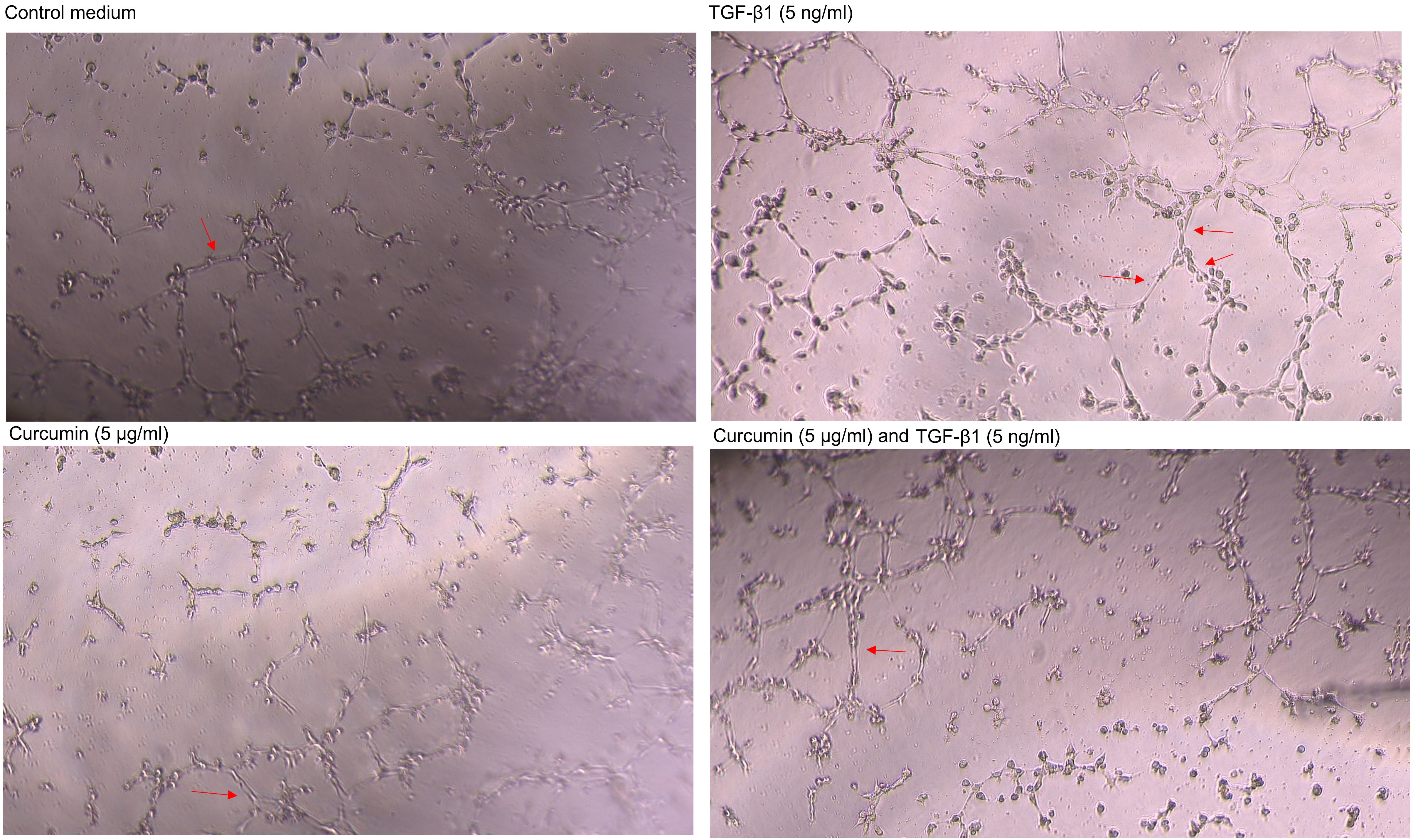
Control medium
TGF-β1 (5 ng/ml)
Curcumin (5 μg/ml)
Curcumin (5 μg/ml) and TGF-β1 (5 ng/ml)

Fig S3.

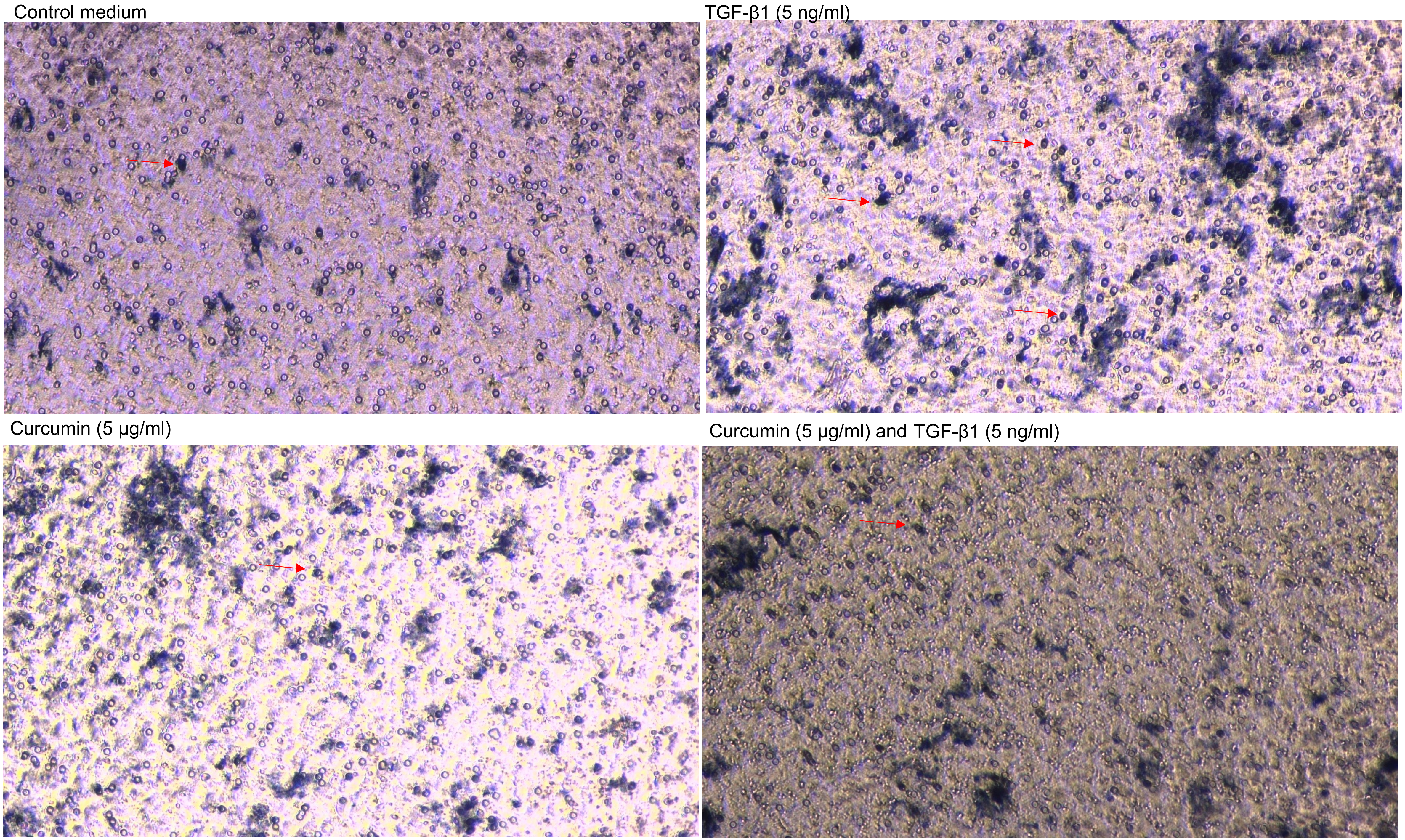
Control medium
TGF-β1 (5 ng/ml)
Curcumin (5 µg/ml)
Curcumin (5 µg/ml) and TGF-β1 (5 ng/ml)

Fig S4.

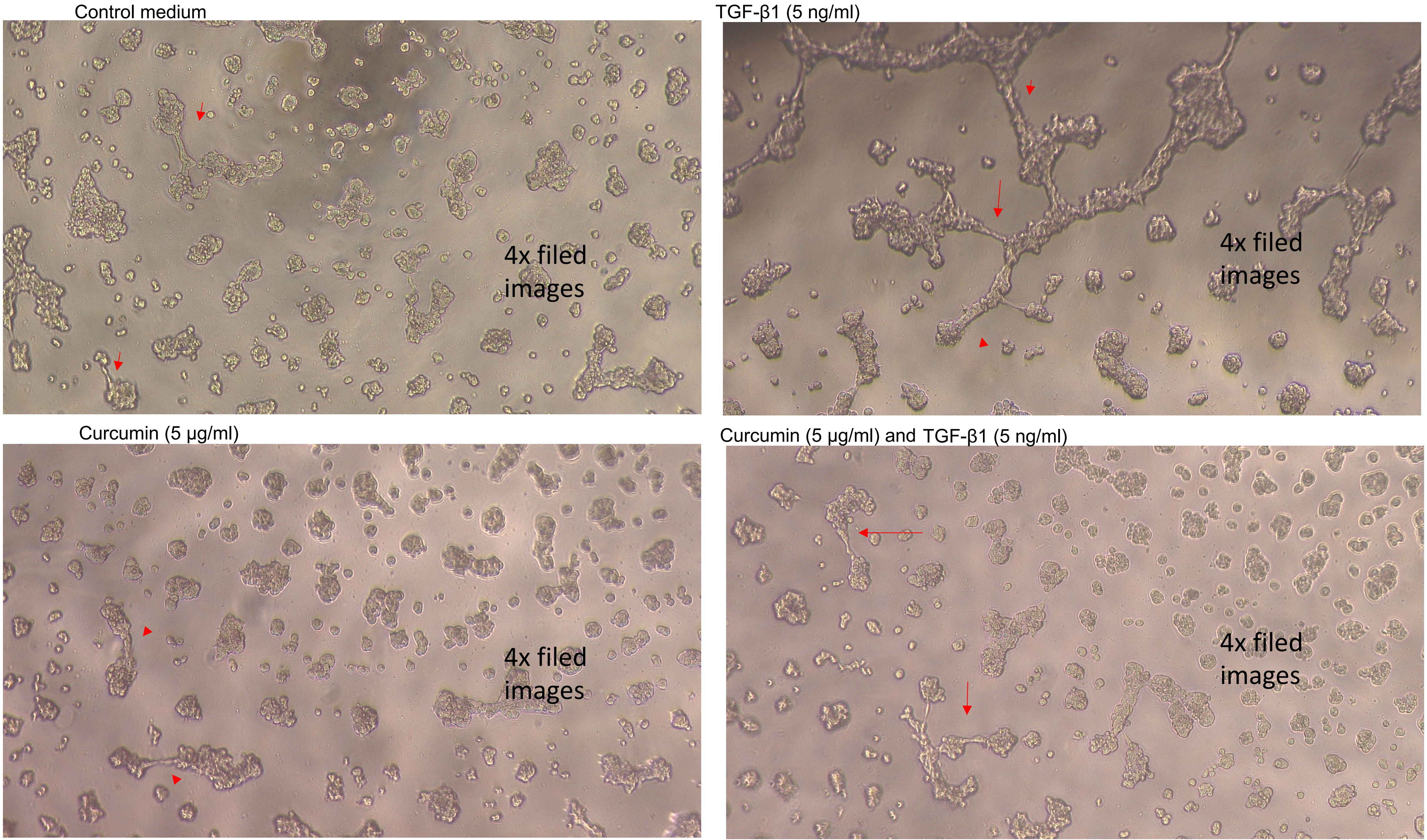
Control medium
4x filed images
TGF-β1 (5 ng/ml)
4x filed images
Curcumin (5 µg/ml)
4x filed images
Curcumin (5 µg/ml) and TGF-β1 (5 ng/ml)
4x filed images

Fig S4.

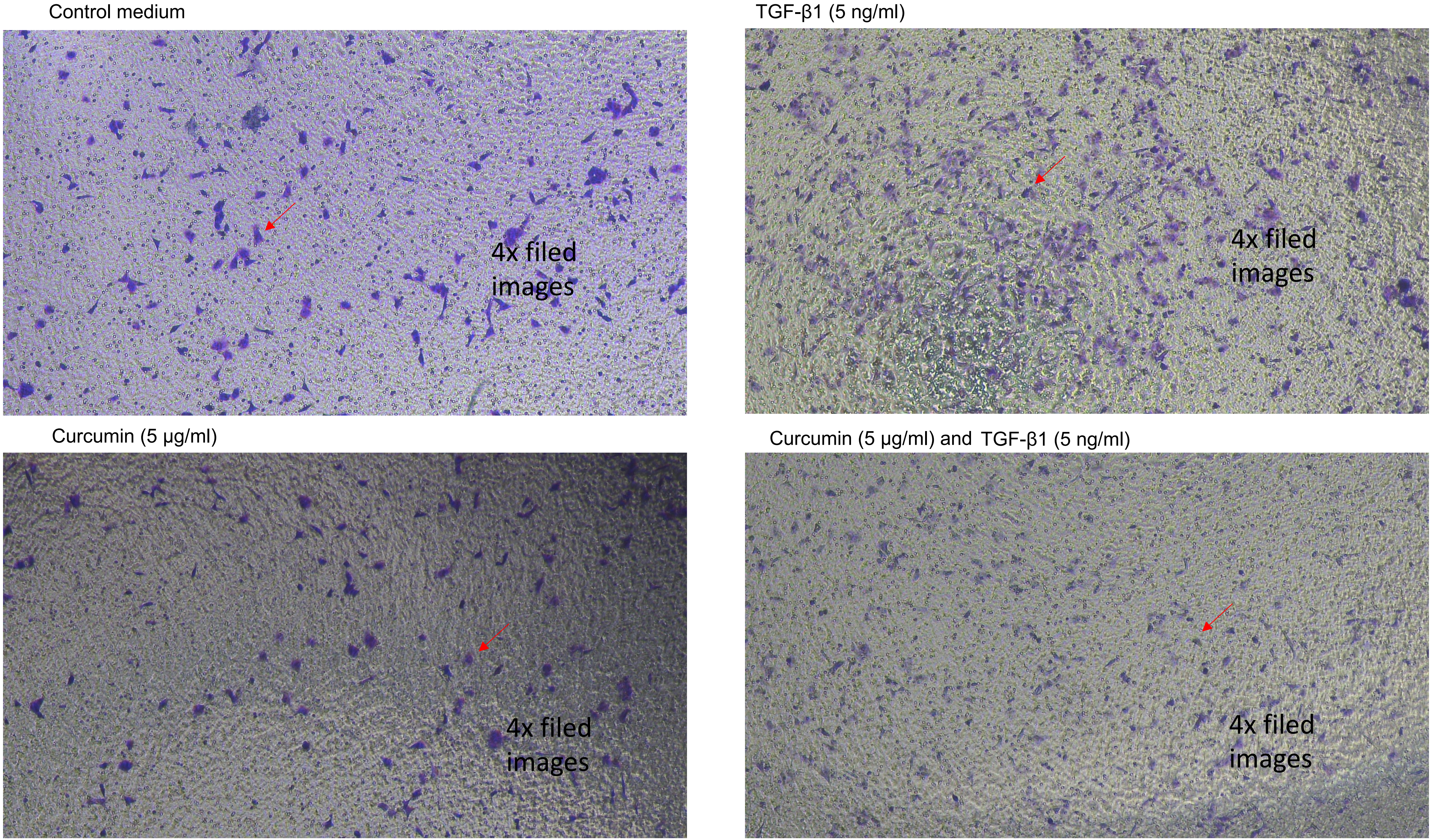
Control medium
4x filed images
TGF-β1 (5 ng/ml)
4x filed images
Curcumin (5 μg/ml)
4x filed images
Curcumin (5 μg/ml) and TGF-β1 (5 ng/ml)
4x filed images
