# Supplementary material for: Curcumin Suppresses TGF-β1-Induced Myofibroblast Differentiation and Attenuates Angiogenic Activity of Orbital Fibroblasts
Source: Int J Mol Sci. 2021 Jun 25;22(13):6829. doi: 10.3390/ijms22136829 (PMC8268269; doi:10.3390/ijms22136829)
Supplement: Supplementary file 1 [file ijms-22-06829-s001.zip › Supplementary Table 1 Rev2 0615.pdf]

Supplementary Table 1

Figure 1A Raw Data

Cell viability 10% FBS-containing medium overnight and treated with TGF-β1 for additional 24 hours. Cells were suspended in 2 ml PBS (Donor 1) or 0.5 ml PBS (Donor 2) for counting.

Donor 1	cells ($1*10^4$) / ml	Donor 2	cells ($1*10^4$) / ml
TGF-β1 0 ng/ml	27	TGF-β1 0 ng/ml	117
	20		94
	19		131
TGF-β1 1 ng/ml	31	TGF-β1 1 ng/ml	135
	53		138
	35		136
TGF-β1 5 ng/ml	23	TGF-β1 5 ng/ml	111
	25		116
	30		112

Figure 1B Raw Data

Cell viability in the basal DMEM medium overnight and treated with TGF-β1 for additional 24 hours. Cells were suspended in 2 ml PBS (Donor 1) or 0.5 ml PBS (Donor 2) for counting.

Donor 1	cells ($1*10^4$) / ml	Donor 2	cells ($1*10^4$)/ ml
TGF-β1 0 ng/ml	16	TGF-β1 0 ng/ml	109
	15		110
	23		112
TGF-β1 1 ng/ml	22	TGF-β1 1 ng/ml	86
	20		89
	17		97
TGF-β1 5 ng/ml	11	TGF-β1 5 ng/ml	102
	17		103
	24		104

Figure 2A Raw Data

Cell viability in 10% FBS-containing medium overnight and treated with curcumin, TGF-β1 or both for additional 24 hours. Cells were suspended in 0.5 ml PBS (Donor 1) or 1 ml PBS (Donor 2) for counting.

Donor 1	cells ($1*10^4$) / ml	Donor 2	cells ($1*10^4$) / ml
Curcumin 0 μg/ml TGF-β1 0 ng/ml	100	Curcumin 0 μg/ml TGF-β1 0 ng/ml	74
	128		61
	80		60
Curcumin 1 μg/ml TGF-β1 0 ng/ml	77	Curcumin 1 μg/ml TGF-β1 0 ng/ml	70
	89		76
	82		63
Curcumin 2.5 μg/ml TGF-β1 0 ng/ml	91	Curcumin 2.5 μg/ml TGF-β1 0 ng/ml	55
	86		72
	84		53
Curcumin 5 μg/ml TGF-β1 0 ng/ml	100	Curcumin 5 μg/ml TGF-β1 0 ng/ml	74
	88		55
	104		51
Curcumin 0 μg/ml TGF-β1 5 ng/ml	104	Curcumin 0 μg/ml TGF-β1 5 ng/ml	77
	125		69
	106		71
Curcumin 1 μg/ml TGF-β1 5 ng/ml	115	Curcumin 1 μg/ml TGF-β1 5 ng/ml	66
	106		54
	101		64
Curcumin 2.5 μg/ml TGF-β1 5 ng/ml	88	Curcumin 2.5 μg/ml TGF-β1 5 ng/ml	52
	82		60
	96		71
Curcumin 5 μg/ml TGF-β1 5 ng/ml	74	Curcumin 5 μg/ml TGF-β1 5 ng/ml	57
	78		61
	71		55

Figure 4A Raw Data

Viability of EA. Hy926 cells treated with condition medium from curcumin or TGF-β1-treated orbital fibroblasts. Cells were suspended in 2 ml PBS for counting. (10% FBS-containing medium overnight to treat curcumin or TGF-β1 for 24 hours)

Donor 1	cells ($1*10^4$) / ml	Donor 2	cells ($1*10^4$) / ml

Curcumin 0 μg/ml TGF-β1 0 ng/ml	18	Curcumin 0 μg/ml TGF-β1 0 ng/ml	13
	21		15
	22		15
Curcumin 5 μg/ml TGF-β1 0 ng/ml	11	Curcumin 5 μg/ml TGF-β1 0 ng/ml	12
	20		13
	20		17
Curcumin 0 μg/ml TGF-β1 5 ng/ml	19	Curcumin 0 μg/ml TGF-β1 5 ng/ml	12
	23		12
	15		15
Curcumin 5 μg/ml TGF-β1 5 ng/ml	22	Curcumin 5 μg/ml TGF-β1 5 ng/ml	11
	20		12
	20		14

Figure 4B Raw Data

The relative tube-branching numbers of EA. Hy926 cells cultured at indicated conditioned medium from orbital fibroblasts. (Branch numbers in a 4x field)

Donor 1	Branches	Donor 2	Branches
Curcumin 0 μg/ml TGF-β1 0 ng/ml	3	Curcumin 0 μg/ml TGF-β1 0 ng/ml	32
	3		30
	4		27
	5		38
	3	Curcumin 5 μg/ml TGF-β1 0 ng/ml	24
Curcumin 5 μg/ml TGF-β1 0 ng/ml	3		34
	1		35
	0		31
	3		30
	0		57
	7		36
Curcumin 0 μg/ml TGF-β1 5 ng/ml	8	Curcumin 0 μg/ml TGF-β1 5 ng/ml	74
	9		62
	12		89
	10		73
	5	Curcumin 5 μg/ml TGF-β1 5 ng/ml	20
Curcumin 5 μg/ml TGF-β1 5 ng/ml	0		21
	1		39
	0		32

	1		27
	0		30
	2		

Supplementary Figure 2 Raw Data

The relative transwell migratory motility of EA. Hy926 cells cultured at indicated conditioned medium from orbital fibroblasts. (Migrated cells in a 10x field)

Donor 2	Migrated cells
Curcumin 0 μg/ml TGF-β1 0 ng/ml	112
	107
	85
	105
	139
	115
Curcumin 5 μg/ml TGF-β1 0 ng/ml	119
	154
	125
	167
Curcumin 0 μg/ml TGF-β1 5 ng/ml	219
	166
	199
	157
	210
	215
Curcumin 5 μg/ml TGF-β1 5 ng/ml	151
	135
	114
	154
	168

Supplementary Figure 3 Raw Data

The relative tube-branching numbers of HMEC-1 cells cultured at indicated conditioned medium from orbital fibroblasts. (Branch numbers in a 10x field) and the relative transwell migratory motility of HMEC-1 cells cultured at indicated conditioned medium from orbital fibroblasts. (Migrated cells in a 10x field)

Donor 2	Branches	Donor 2	Migrated cells

Curcumin 0 μg/ml TGF-β1 0 ng/ml	5
	7
	2
Curcumin 5 μg/ml TGF-β1 0 ng/ml	2
	2
	1
Curcumin 0 μg/ml TGF-β1 5 ng/ml	10
	14
	11
Curcumin 5 μg/ml TGF-β1 5 ng/ml	6
	2
	4

Curcumin 0 μg/ml TGF-β1 0 ng/ml	59
	50
	22
	86
	79
	71
Curcumin 5 μg/ml TGF-β1 0 ng/ml	75
	53
	43
	40
	46
	45
Curcumin 0 μg/ml TGF-β1 5 ng/ml	112
	105
	115
	95
	125
	99
Curcumin 5 μg/ml TGF-β1 5 ng/ml	44
	33
	48
	64
	70
	50
